# Blood Nanoparticles – Influence on Extracellular Vesicle Isolation and Characterization

**DOI:** 10.3389/fphar.2021.773844

**Published:** 2021-11-10

**Authors:** Marija Holcar, Maša Kandušer, Metka Lenassi

**Affiliations:** Institute of Biochemistry and Molecular Genetics, Faculty of Medicine, University of Ljubljana, Ljubljana, Slovenia

**Keywords:** extracellular vesicles, blood nanoparticles, contaminants, isolation methods, characterization methods

## Abstract

Blood is a rich source of disease biomarkers, which include extracellular vesicles (EVs). EVs are nanometer-to micrometer-sized spherical particles that are enclosed by a phospholipid bilayer and are secreted by most cell types. EVs reflect the physiological cell of origin in terms of their molecular composition and biophysical characteristics, and they accumulate in blood even when released from remote organs or tissues, while protecting their cargo from degradation. The molecular components (e.g., proteins, miRNAs) and biophysical characteristics (e.g., size, concentration) of blood EVs have been studied as biomarkers of cancers and neurodegenerative, autoimmune, and cardiovascular diseases. However, most biomarker studies do not address the problem of contaminants in EV isolates from blood plasma, and how these might affect downstream EV analysis. Indeed, nonphysiological EVs, protein aggregates, lipoproteins and viruses share many molecular and/or biophysical characteristics with EVs, and can therefore co-isolate with EVs from blood plasma. Consequently, isolation and downstream analysis of EVs from blood plasma remain a unique challenge, with important impacts on the outcomes of biomarker studies. To help improve rigor, reproducibility, and reliability of EV biomarker studies, we describe here the major contaminants of EV isolates from blood plasma, and we report on how different EV isolation methods affect their levels, and how contaminants that remain can affect the interpretation of downstream EV analysis.

## Introduction

Biological fluids are an ideal source for early disease discovery and for monitoring of disease progression or the success of a treatment (Alix-Panabières and Pantel, 2017; Bracht et al., 2018; Morrison and Goldkorn, 2018). The most widely used and studied source of disease biomarkers is peripheral circulating venous blood, as its collection is minimally invasive and can be performed repeatedly (González and Falcón-Pérez, 2015). Blood has an important role in the transmission of information between cells and tissues, and it can accumulate diverse signals as a result of disease or distress, in the form of nucleic acids, proteins, metabolites, and extracellular vesicles (EVs). Blood will thus reflect the health of the organs and tissues.

Extracellular vesicles are nanometer-to micrometer-sized spherical particles (diameter, 40–1,000 nm; density, 1.110–1.190 g/cm^3^) that are enclosed by a phospholipid bilayer; they can be secreted by most cell types, and they cannot replicate (Mathieu et al., 2019). EVs are very heterogeneous in size, biophysical characteristics, molecular content, function, biogenesis, and release pathways (Zaborowski et al., 2015; Zhang Q. et al., 2019; Dong et al., 2020). Based on their sizes and cellular origins, EVs released by living cells can be split into two major groups: exosomes (diameter, 50–150 nm), which originate from multivesicular bodies, and microvesicles (diameter, ≤1 µm), which bud from the plasma membrane. However, the cellular origins of EVs are difficult to ascertain using simple methods, such as measurements of size, and even more so *in vivo*; e.g., for EVs in body fluids after they have already been released into the systemic circulation (Karasu et al., 2018). Therefore, the Minimal Information for the Study of EVs (MISEV 2018) guidelines have proposed the use of descriptive terms for EV subtypes that refer to their physical characteristics, such as size (diameters: <100 nm, small EVs; 100–200 nm, medium/large EVs; >200 nm, large EVs) or density (low, medium, high densities, with each range defined) (Théry et al., 2019). Importantly, the composition and the physiological state of the cell of origin is reflected by the molecular cargo of EVs, as these can contain functional nucleic acids (e.g., mRNAs, miRNAs, DNA), proteins (e.g., cytoskeletal proteins, tetraspanins, integrins), and specific enrichment of molecules typical of lipid rafts (e.g., ceramide, cholesterol, phosphatidylserine) (Ghosh et al., 2014; Doyle and Wang, 2019; Jeppesen et al., 2019).

Extracellular vesicles used to be regarded as merely a disposal mechanism used by cells, or even as “cellular dust”. However, they can also cross extracellular space and even biological barriers, and they can activate specific pathways or directly transfer biological contents upon binding to their target cells, which indicates that they can influence the physiology of recipient cells and tissues. EVs are important mediators of adaptations to micro-environmental changes, and they participate in intercellular communication and immune responses (Clotilde et al., 2009; Karasu et al., 2018; Shao et al., 2018). Therefore, EVs represent a promising source of novel diagnostic and prognostic biomarkers, particularly in terms of their traceability to their cell type or tissue origin and its physiological state, their stability, their protection of their internal cargo from degradation in the extracellular environment, and their presence in circulating blood at estimated concentrations of 10^10^ EV/ml (Dickhout and Koenen, 2018; Johnsen et al., 2019; Vasconcelos et al., 2019; Holcar et al., 2020; Hoshino et al., 2020; Kamal and Shahidan, 2020; Laurenzana et al., 2021). Even more importantly, in the future, should they be modified to encapsulate different drugs, they can potentially be used as new therapeutic options for a nonimmunogenic delivery system with high target specificity upon systemic administration (Dang et al., 2020).

Molecular components of blood EVs have generally focused on proteins and miRNAs, and these have already been studied in the context of many diseases, as they might represent easily detectable and disease-specific biomarkers. The presence and concentrations of specific EV-associated proteins have been analyzed in many different diseases. Particular examples can be seen for cancers: the correlation of baseline EV-related PD-L1 with tumor response to treatment for melanoma; dysregulation of tumor-associated antigens (including BAGE, PD-L1, MAGE-3, AKAP4) in EVs of patients with nonsmall-cell lung cancer; higher expression of LZH8, HER2 and PSA in EVs from lymphoma patients; and even separation of tumor-derived EVs from those derived from healthy tissue according to their contents of VCAN, TNC, and THBS2 (Liu et al., 2019; Wu et al., 2019; Cordonnier et al., 2020; Hoshino et al., 2020). In neurodegenerative diseases, the amyloid-β and tau proteins have been detected in brain-secreted EVs from blood from patients with Alzheimer’s disease, and similarly for the α-synuclein protein in patients with Parkinson’s disease (Badhwar and Haqqani, 2020; Yu et al., 2020). In the autoimmune disease rheumatoid arthritis, patients with more IgM rheumatoid-factor-positive EVs have higher disease activity (Arntz et al., 2018). Higher levels of CD31 and annexin A5 have been reported for EVs of patients who later develop major adverse cardiovascular and cerebral events, which also indicates the involvement of EVs in cardiovascular diseases (Johnsen et al., 2019).

As well as such changes in their molecular composition in blood, the EV concentrations and size profile might be linked to physiological factors like hypoxia, autophagy or stress, and hence be typical for a diverse set of diseases (Vasconcelos et al., 2019). Indeed, increases in plasma EV levels have been reported in connection with several cancers (Navarro et al., 2019; Badovinac et al., 2021), and also for cardiovascular and autoimmune diseases (Dickhout and Koenen, 2018; Maione et al., 2020).

**FIGURE 1:**
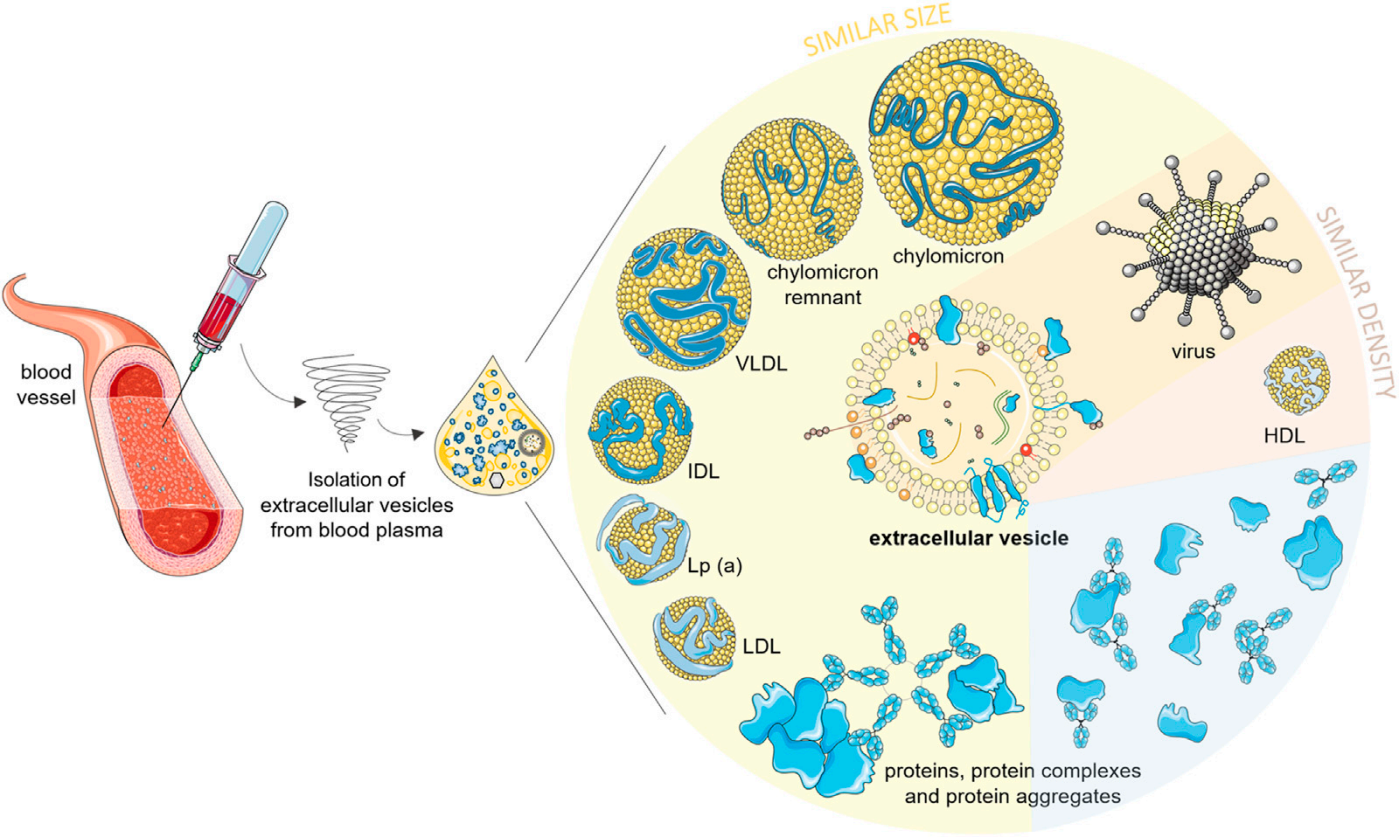
Possible nano-sized contaminants in an extracellular vesicle isolate of blood plasma. Lipoproteins larger than high density lipoproteins (HDLs) and larger protein aggregates/complexes are similar to EVs in size. HDLs have similar density, and viruses can be of similar density and size as EVs. Soluble proteins are smaller and denser than EVs, but they can form a protein biocorona around EVs or aggregate with any other nanoparticles during the isolation procedures, which will also contribute to contamination of EV isolates. EVs, extracellular vesicles; HDLs, high density lipoproteins; LDL, low-density lipoproteins; Lp(a), lipoprotein a; ILDs, intermediate-density lipoproteins; VLDLs, very low density lipoproteins. Part of this figure was modified from SMART (Servier Medical Art), licensed under a Creative Common Attribution 3.0 Unported License. http://smart.servier.com/.

Despite great interest in EVs as biomarkers of pathologies, it remains challenging to entirely separate EVs from other blood nanoparticles, such as proteins and lipoproteins, and potentially viruses, due to their overlapping characteristics (Figure 1; Table 1). Consequently, isolation and downstream analysis of EVs isolated from blood plasma remain a unique challenge (Bracht et al., 2018; Freitas et al., 2019; Geeurickx and Hendrix, 2020; Nieuwland et al., 2020), with important impact on biomarker study outcomes. To help to improve rigor, reproducibility, and reliability of EV biomarker studies, we describe here the major contaminants of EV isolates from blood plasma, and we report on how different EV isolation methods can affect their levels, and how remaining contaminants can affect the interpretation of downstream EV analysis.

**TABLE 1:** Characteristics of blood plasma nanoparticles.

Blood-plasma nanoparticle	Subgroup	Diameter (nm)	Density (g/cm^3^)	Concentration in blood plasma (particles/mL)	Detection in extracellular-vesicle isolates	References
Extracellular vesicles (EVs)	All plasma EVs	40–1,000	1.08–1.21 (1.110–1.190)	10^8^–10^13^ (on average, 10^10^)	Presence/concentration of EV-derived proteins (e.g., tetraspanins CD9, CD63, CD81, TSG101, Flotilin1, others)	Simonsen, (2017); Berckmans et al. (2019); Johnsen et al. (2019); Mathieu et al. (2019); Tian et al. (2020)
	Platelet-derived EVs			10^8^–10^10^	Presence/concentration of platelets in plasma prior to EV isolation; Presence/concentration of CD41, CD42a, CD61, CD62p EVs in EV isolate	
Lipoproteins	High density	5–12	1.063–1.210	10^16^ ^a^	Presence/concentration of ApoA1	Nakajima et al. (2001); Wojczynski et al. (2011); Sabaka et al. (2013); Tsimikas et al. (2018); Tian et al. (2020)
	Low density	18–25	1.019–1.063	10^15^ ^a^	Presence/concentration of ApoB100	
	Intermediate density	25–35	1.006–1.019	10^12^ ^a^ ^,^ ^b^	Presence/concentration of ApoB100	
	Lipoprotein (a)	12–500	1.048–1.086	10^12^ ^b^	Presence/concentration of ApoB100 and ApoA1	
	Very low density	30–80	0.930–1.006	10^12^ ^a^ ^,^ ^b^	Presence/concentration of ApoB100	
	Chylomicrons	75–1,200	<0.930	10^13^ ^a^ ^,^ ^c^	Presence/concentration of ApoB48	
	Chylomicron remnants	30–80	0.950–1.006	10^12^–10^13^ ^a^ ^,^ ^c^ ^,^ ^d^	Presence/concentration of ApoB48		
Protein aggregates	-	<1–15,000	∼1.4 (dense packing)	10^17^ ^b^ of albumin 10^16^ ^b^ of globulins	Protein concentration (absorbance at 280 nm, bicinchoninic acid/Bradford assay)	Buis et al. (1996); Stanyon and Viles, (2012); Simonsen, (2017)
Viruses	-	30–300	1.16–1.18 (most retroviruses)	Depends on the infection status	Presence of viral genome (DNA/RNA extraction and quantification)	Nolte-‘t Hoen et al. (2016), Raab-Traub and Dittmer, (2017)

a Numbers can change significantly with prandial status and diet composition.

b Numbers of particles calculated from reported mass concentrations in plasma.

c Mostly present post-prandially.

d Numbers of particles calculated from reported mass concentrations of their specific protein (ApoB48) in plasma.

## Contaminants of Extracellular Vesicle Isolates From Blood Plasma and Their Relevance for Extracellular Vesicle Isolation and Characterization

Blood is the most commonly used body fluid for liquid biopsies – a term that refers to the assessment of biomarkers in biological fluids, as a minimally invasive alternative to tissue biopsies. EVs are emerging as promising new biomarkers in liquid biopsies; however, blood plasma is a very complex biofluid (Lötvall et al., 2014; Lu et al., 2019; Notarangelo et al., 2019; Ruhen and Meehan, 2019; Vasconcelos et al., 2019; Pang et al., 2020). The abundance of different types of plasma nanoparticles thus leads to challenges for EV isolation and characterization.

In the context of diseases, the main plasma nanoparticles of interest are EVs, which can accumulate in the blood after their release from specific pathological tissues, while the blood itself also contains a variety of cells that release EVs. It has been shown that up to 30% of blood EVs are erythrocyte-derived EVs, and up to 20% are leucocyte-derived EVs (Berckmans et al., 2019). Platelets are an anuclear component of blood, and these require special attention as **platelet-derived EVs** are the most abundant EVs in human blood, as they represent 50–90% of all circulating large EVs in healthy subjects (Table 1) (Berckmans et al., 2019; Taus et al., 2019). Their elevated concentrations in blood are linked to vascular diseases and even to some types of cancers, and thus these can carry important clinical information (González and Falcón-Pérez, 2015). On the other hand, due to the important role of platelets in the process of thrombosis, these are particularly susceptible to activation during blood collection and handling. This can lead to abundant *ex-vivo* platelet vesiculation, especially if there is a delay between sample collection and processing (Brahmer et al., 2019; Taus et al., 2019; Tripisciano et al., 2020; Antich-Rosselló et al., 2021; Puhm et al., 2021). This uncontrolled release of nonphysiological EVs can adversely affect downstream EV analysis; e.g., it has been shown that contaminating platelet-derived EVs can skew the isolated miRNAs populations in patient and control samples (Palviainen et al., 2020). Further important pre-analytical factors that can affect isolation and characterization of EVs include size of the needle used to draw the blood, correct handling of blood samples, and prompt and complete separation of plasma from uncoagulated blood cells and platelets (i.e., to limit their activation). The purity of EV preparations can be evaluated by flow cytometric quantification of CD41^+^, CD42^+^ or CD62P + nanoparticles, which are characteristic of platelet-derived EVs (Pugholm et al., 2016; Berckmans et al., 2019; Brahmer et al., 2019).

**Proteins** and protein aggregates are the most common nonEV contaminants of EV preparations from blood, and these can considerably impact the downstream analyses (Table 1) (Yuana et al., 2014; Sódar et al., 2016; Takov et al., 2019; Théry et al., 2019). Blood plasma contains approximately 60 mg/ml to 80 mg/ml protein, with wide ranges of concentrations of different proteins (i.e., pg/mL to mg/mL) and a vast heterogeneity of their glycosylation profiles, as up to 50% of plasma proteins are glycosylated (Anderson and Anderson, 2002; Shamsi et al., 2012). About 50–60% of all plasma proteins are albumins, and 40% are globulins, of which 10–20% are immunoglobulins G (Leeman et al., 2018). Coagulation factors are the next most abundant proteins in the blood (e.g., fibrinogen, 4%), followed by lipoproteins (1%) and iron-binding/transferring proteins (1%), with <1% of the total circulating proteins represented by different hormones, lysosomal proteins, proteins released from dead or damaged cells, proteins related to diseases or infection (e.g., cytokines, components of the complement), and also biotherapeutic proteins used as drugs (Karimi et al., 2018; Geyer et al., 2019; Pietrowska et al., 2019). These proteins can all contribute to the formation of protein aggregates, which can have similar biophysical properties as EVs, such as size, charge, and buoyant density, and which can thus co-isolate and contaminate EV preparations (Sódar et al., 2016; Simonsen, 2017; Johnsen et al., 2019). The concentration of cell-free nucleic acids can also increase in correlation to different diseases such as cancers, autoimmune diseases, and inflammatory reactions (Endzelinš et al., 2017; Zhang L. et al., 2019; Duvvuri and Lood, 2019). These cell-free nucleic acids and the proteins bound to them can also form insoluble aggregates in the blood (Duvvuri and Lood, 2019).

Recent studies on EVs and previous studies on nanoparticles of nonbiological origin have suggested that the same sets of proteins can bind physiologically to the surface of EVs in body fluids to form the protein corona, which thus coats the EVs (Palviainen et al., 2019; Priyanka et al., 2020). The composition of the protein corona depends on the composition of the biofluid and its protein concentration, on the fluid conditions (i.e., static vs. flowing), and on the temperature and nanoparticle properties (Nguyen and Lee, 2017). In blood, the EV protein corona typically consists of immunoglobulins, complement proteins, coagulation factors, cytokines, enzymes, DNA, and RNAs (Cvjetkovic et al., 2016; Buzás et al., 2018). When blood proteins are part of the protein corona, they can have notable effects on EVs, including their mobility, interactions with their surroundings or target surfaces, and recognition by the immune system, which will affect the physiological role of the EVs (Strojan et al., 2017; Buzás et al., 2018; Charoenviriyakul et al., 2018; Skliar et al., 2018; Ezzat et al., 2019). It is therefore important to efficiently remove unbound blood proteins from EV isolates, while still appreciating the physiological roles of blood proteins that are bound to EVs as the protein corona. The purity of EV preparations can also be evaluated by determination of the nanoparticle-to-protein ratio or the nanoparticle-to-lipid ratio (Théry et al., 2019).

Another common contaminant of blood EV isolates are the **lipoproteins** (Table 1). These are spherical particles that transport the major lipids in the bloodstream of humans throughout the body. They consist of an amphipathic surface of protein(s), free cholesterol, and phospholipids, which surround a hydrophobic core that contains cholesterol esters and triacylglycerols (Ference et al., 2020). Depending on the lipid and protein contents, these circulating lipoproteins are separated into high density lipoproteins (HDLs), low density lipoproteins (LDLs), intermediate density lipoproteins (IDLs), very low density lipoproteins (VLDLs), lipoprotein a (Lp(a)), chylomicrons, and chylomicron remnants (Table 1). Lipoproteins can be identified based on their sizes, chemical compositions, physicochemical and flotation characteristics, and electrophoretic mobilities (Nakajima et al., 2001; Feingold and Grunfeld, 2010; Pasquetto et al., 2016; Karimi et al., 2018). Importantly, lipoproteins share either similar sizes or densities with EVs (Figure 1; Table 1) (Karimi et al., 2018; Johnsen et al., 2019). In blood, these lipoproteins are at concentrations far higher than those of EVs (Johnsen et al., 2019). They also fluctuate intra- and inter-individually, and are significantly affected by prandial status (Varga et al., 2014; Simonsen, 2017). Additionally, HDLs and LDLs have both been shown to transport miRNAs, which can co-isolate with EV-associated RNAs (Vickers et al., 2011; Mateescu et al., 2017). Also, LDLs added to pure EV preparations can associate with the EV surface *in vitro*, and lipoprotein–EV interactions appear to even have roles in the pathogenesis of atherothrombosis, which highlights the likelihood of lipoprotein co-isolation with EVs (Sódar et al., 2016; Chiva-Blanch and Badimon, 2019). It is thus important to take special care to remove lipoproteins from EVs in samples before downstream analysis, and to sufficiently test for lipoprotein contamination in EV isolates. The purity of EV preparations can be evaluated by determining the apolipoprotein concentrations (ApoA1 for HDLs; ApoB, ApoE for other lipoproteins) in samples by enzyme-linked immunosorbent assays (ELISA) or Western blotting (Dong et al., 2020; Matsumoto et al., 2020; Zhang et al., 2020; Ji et al., 2021).

In the context of infectious diseases, infectious agents like **viruses** can also co-isolate with blood EVs (Table 1). Viral particles share similar biophysical properties with EVs, such as size, molecular composition, and physical features (Martin-Jaular et al., 2021). For example, enveloped viruses can share several biogenesis pathways with EVs, such as seen for Human immunodeficiency virus and Hepatitis C virus. This results in secretion of diverse types of nanoparticles from infected cells, such as naked virions, EVs containing infective viral genomes and quasi-enveloped viruses, classical complete viral particles, and also EVs modified by the infection and EVs that are not altered by the infection (Nolte-‘t Hoen et al., 2016; Sódar et al., 2016; Ramirez et al., 2018; Martins and Alves, 2020; Martin-Jaular et al., 2021). Even in cases of nonenveloped viruses (e.g., Hepatitis A virus), EVs can provide an ‘envelope’ for the viruses and thus propagate infection to other cells (Nolte-‘t Hoen et al., 2016). Consequently, the separation of host EVs from any virions is extremely challenging, and at present this is limited to affinity-based purification strategies (Jung et al., 2020; Martin-Jaular et al., 2021). It is important to note that isolation of EVs from biological samples of patients with viral infections can also present safety risks for laboratory operators, which was indeed highlighted by the recent COVID-19 pandemic that was caused by Severe acute respiratory syndrome coronavirus 2 (SARS-CoV-2). SARS-CoV-2 infection primarily affects the upper respiratory tract and lungs, but the virus can also be detected in blood (Nunez Lopez et al., 2021). Even though additional treatments of clinical samples prior to EV isolation from blood can have a profound impact on their isolation and related contaminants, inactivation of SARS-CoV-2 by heat or solvent–detergent treatments is recommended (Jureka et al., 2020; Frigerio et al., 2021).

Finally here, bacterial EVs have also been found and quantified in human plasma samples (Tulkens et al., 2020). Collected, non-sterile blood samples can also be contaminated with fungi, which release fungal EVs that can co-purify with blood EVs (Sódar et al., 2017). It is, therefore, important to evaluate the possible biological contaminants of all body fluid samples also from the safety perspective, to eliminate or minimize the potential risk to laboratory personnel during the handling of samples. The presence of microbial contaminants in blood and EV isolates can be evaluated by checking for their genetic material using PCR.

To conclude, blood contains diverse nanoparticles besides EVs, which include proteins, lipoproteins and viruses. As these can be similar to EVs in terms of certain characteristics, they can be co-isolated with EVs from blood. It is thus important to select method(s) for isolation of EVs from blood that are compatible with the planned downstream analysis, and to determine the levels of contaminants in EV preparations. This is also highly encouraged by the recent guidelines for minimal information for studies on EVs, as supported by the International Society for Extracellular Vesicles (Théry et al., 2019). However, to improve the reliability and reproducibility of EV biomarker studies there remain important challenges to be overcome in terms of how preanalytical variables can affect the levels of nonEV nanoparticles in blood samples, and how these nonEV nanoparticles might affect EV isolation and downstream analysis.

## The Selection of Isolation Method Affects the Purity of Extracellular Vesicle Isolation From Blood

Extracellular vesicles can be isolated from biological fluids using diverse methods according to their sizes, densities, charges, or specific markers, such as size-exclusion chromatography (SEC), ultrafiltration, asymmetric flow field-flow fractionation (AF4), ultracentrifugation (over density gradients), precipitation, and immunoaffinity approaches (Witwer et al., 2013; Ramirez et al., 2018; Gandham et al., 2020). For in-depth descriptions of various isolation methods, please see previous publications (Monguió-Tortajada et al., 2019; Cocozza et al., 2020; Sidhom et al., 2020). The selection of the EV isolation method also influences the co-isolation of other blood nanoparticles with similar properties. Therefore, the choice of the method used is crucial, and should be made with the downstream analyses in mind. Table 2 provides a summary of the characteristics of the commonly used methods for isolation of EVs from human plasma.

**TABLE 2:** Characteristics of the most commonly used methods for isolation of extracellular vesicles (EVs) from human plasma.

Characteristic	Size exclusion chromatography	Ultrafiltration	Differential ultracentrifugation	Density gradient ultracentrifugation	Precipitation	Immunoaffinity purification	Asymmetric flow field-flow fractionations	Microfluidics	References
**Plasma volume (ml)**	≤0.5	≥3.0	≥3.0	0.5–3.0	0.5–3.0	≤0.5 ml	≤0.5 ml	≤0.5 ml	Dong et al. (2020); Veerman et al. (2021)
**Time (h)**	1.5–2.0	0.5	3.0–4.0	16.0–90.0	2.0–16.0	4.0–overnight (without coupling of beads)	<1.0	<1.0	Coumans et al. (2017); Doyle and Wang, (2019); Monguió-Tortajada et al. (2019); Patel et al. (2019); Zhang and Lyden, (2019)
**Cost**	High	Medium	Low	Low	Low	High	Medium	Very high	Doyle and Wang, (2019); Sidhom et al. (2020)
**Principle of isolation**	Difference in hydrodynamic size and shape between particles; particles flow through or around the stationary phase; larger particles elute first	Difference in hydrodynamic size between particles; particles larger than cut-off size of membrane retained in concentrate	Difference in sedimentation coefficient (hydrodynamic size and density) of particles; particles with different sedimentation coefficients pellet at different centrifugation speeds	Difference in sedimentation and flotation coefficient of particles; during centrifugation particles distribute through density gradient matrix according to sedimentation and flotation index	Difference in charge/solubility between particles, leading to precipitation	Interaction between specific protein and antibody; antibodies often coupled to magnetic beads and bound particles separated using magnetic separation	Difference in hydrodynamic size of particles, leading to differential transport velocity of particles in laminar flow profile according to particle position above the semipermeable membrane	Separation on microchip; combination of different approaches	Zhang and Lyden, (2019); Gandham et al. (2020)
**Nanoparticles efficiently removed**	Soluble proteins, high density lipoproteins	Soluble proteins	None	Larger lipoproteins, protein aggregates (depends on gradient)	None	All particles except protein of interest	Soluble proteins; also lipoproteins (depends on protocol)	Depends on isolation method(s) used	Bo et al. (2014); Vergauwen et al. (2017); Wu et al. (2020)
**Major contaminants in EV isolates** **^a^**	Lipoproteins of similar size to EVs	Lipoproteins of similar size or larger than EVs, larger protein aggregates	Protein aggregates, aggregates of proteins and cell-free nucleic acids, lipoproteins	High density lipoproteins (depends on gradient and protocol)	Lipoproteins, soluble proteins, complexes of proteins and cell-free nucleic acids	Nonspecific binding of abundant plasma proteins	Lipoproteins of similar size to EVs (efficiency depends on programmable cross-flow intensity)	Depends on isolation method(s) used	Gandham et al. (2020); Holcar et al. (2020); Tian et al. (2020)
**Optimization of critical steps**	Use fasting plasma; optimize eluted fractions concentrated into final sample	Membrane type (regenerated cellulose, pore size 10 kDa best for centrifugal ultracentrifugation)	Number of ultracentrifugation steps, acceleration, rotor, solution viscosity, duration	Top *versus* bottom loading, gradient medium, gradient used	N.A.	Elution of sample from antibodies	Optimize cross-flow velocity and channel thickness	N.A.	Sitar et al. (2015); Mørk et al. (2016); Vergauwen et al. (2017); Sidhom et al. (2020)
**Frequency of use as primary EV isolation method** **^b^ **^,^ **^c^**	Used exclusively, it is the third most often used method; frequency of use is increasing; can be used as a first step in combined protocols, followed by other methods	Not used on its own	Used exclusively, it is the most often used method; frequently used in combination with ultrafiltration and precipitation	Usually used in combination with other methods	Used exclusively, it is the second most often used method; frequently used with differential ultracentrifugation	Used commonly, if starting plasma volume <1.0 ml	Low, use is increasing in last years	Low, use is increasing in last years	Gardiner et al. (2016); Royo et al. (2020)
**Frequency of use as additional clean-up/purification method** **^b^**	Third most often used method	Frequently used to concentrate samples (e.g., after size-exclusion chromatography, before density gradient ultracentrifugation)	The most commonly used method (ultracentrifuge wash)	Second most often used method	Often follows differential ultracentrifugation	D.N.R.	Can be used for further separation of pre-isolated EV samples, usually coupled to different detectors	D.N.R.	Sitar et al. (2015), Sitar et al. (2017); Gardiner et al. (2016); Holcar et al. (2020); Royo et al. (2020); Tian et al. (2020)
**Concentration of isolated particles (particles/mL plasma)**	1.4 × 10^11^ ^d^–6.5 ×10^11^ ^e^	1.5 ×10^12^ ^e^ – 3.2 × 10^12^ ^d^	4.9 × 10^8^ ^d^–1.5 ×10^12^ ^e^	1 ×10^10^ ^e^	1.4 ×10^11^–5.5 × 10^12^ ^d,e^	1 ×10^8^ ^e^	1.1 ×10^1^°F	N.A.	Sitar et al. (2015); Askeland et al. (2020); Dong et al. (2020); Tian et al. (2020); Wei et al. (2020); Veerman et al. (2021)
**Recovery efficiency (%)**	100^e^; 65^d^	80–84^e^; 37^d^	5.0–22^e^; 40 (for single step); ∼16 (for twice at 1,000,00× *g*)^d^	30 (using iodixanol gradient)^e^	56^e^	>90^e^	5-fold that of differential ultracentrifugation^g,h^; 90^i^	82^j^	Kol et al. (2010); Lobb et al. (2015); Busatto et al. (2018); Chen et al. (2019); Geeurickx et al. (2019); Dong et al. (2020) Marioli and Kok, (2020); Pang et al. (2020)
**Purity of EV isolate (%)**	28	11	78	Similar to differential ultracentrifugation	5–19	Greater than differential ultracentrifugation	D.N.R.	N.A.	Lobb et al. (2015); Tian et al. (2020)
**Functionality of isolated EVs**	Good	Medium	Medium	Good	Medium	Poor	D.N.R.	Depends on isolation method(s) used	Sidhom et al. (2020)
**Typical markers analyzed in samples**	EV protein and miRNA cargoes	Depends on combined isolation method	Used for every marker	Morphology of EVs and EV protein cargoes	EV RNA cargoes	EV protein and miRNA cargoes	Depends on coupled detector(s)	Depends on isolation method(s) used	Coumans et al. (2017); Chen et al. (2020); Gaspar et al. (2020); Pang et al. (2020); Sidhom et al. (2020)
**Prevalent end-point method/application**	Flow cytometry; functional studies	*In-vivo* and *in-vitro* functional studies	*In-vivo* and *in-vitro* functional studies	Flow cytometry; proteomics; basic science of EV heterogeneity and biology; translational studies	RNA analysis	Flow cytometry and proteomics	D.N.R.	Depends on isolation method(s) used	Nordin et al. (2015) Gardiner et al. (2016) Pang et al. (2020)
**Analysis methods affected by major contaminants** **^k^**	Methods for label-free quantification of EVs; RNA analysis	Depends on combined isolation method	Methods for label-free quantification of EVs; proteomics	N.A.	Methods for label-free quantification of EVs; electron microscopy; DNA/RNA analysis; mass spectrometry	Methods for label-free quantification of EVs; atomic force microscopy; functional studies^l^	Methods for label-free quantification of EVs	Depends on isolation method(s) used	Nordin et al. (2015); Guerreiro et al. (2018); Doyle and Wang, (2019)

a Except for immunoaffinity precipitation, if viral particles are present, they will remain as a contaminant after all of the isolation protocols (Jung et al., 2020; Martins and Alves, 2020).

b Frequency of use for each method when starting material is plasma is estimated based on two worldwide studies conducted in 2016 and 2020 by ISEV; for exact frequency of use combining all types of starting materials, see Gardiner et al. (2016) and Royo et al. (2020).

c Based on EV-TRACK data (search parameters: biofluid: blood plasma, species: *Homo sapiens*) at http:evtrack.org, collected to 14 October 2021.

d Measured by nano-flow cytometry.

e Measured by nanoparticle tracking analysis.

f Measured by multi-angle light scattering detection.

g EVs, isolated from cultured media, not blood plasma.

h Measured by tunable resistive pulse sensing.

i Measured and calculated from analyzing polystyrene latex nanoparticles in phosphate-buffered saline.

j EVs, isolated directly from full blood.

k Special care should be taken when analyzing samples without further EV, sample processing.

l Functional EV, studies require EV, samples to be devoid of antibody contaminants.

N.A., not applicable; D.N.R., data not reported.

### Extracellular Vesicle Isolation Based on Particle Size

#### Size-Exclusion Chromatography

As for ultrafiltration (see below), SEC is used to isolate EVs based on their sizes. In SEC, the particles move through the fixed stationary phase (beads with pores with specific diameters) with the fluid flow, either under gravity or under a small differential pressure (Taylor and Shah, 2015). Any particle that is small enough to enter the bead pores is delayed due to its increased path length. Instead, particles that are larger than the pore diameter cannot enter the beads, and thus travel along with the void volume of the fluid (Bo et al., 2014). Isolation of EVs from different body fluids by SEC usually leads to EV preparations that are free from significant protein contamination, where the EVs retain their structures and physiological functions, accompanied by very high vesicle yields (Baranyai et al., 2015; Gámez-Valero et al., 2016; Geeurickx et al., 2019). However, low levels of albumin contamination of such EV isolates have been reported (Baranyai et al., 2015; An et al., 2018). While SEC is mostly successful in removing HDLs, other lipoproteins of similar sizes to EVs (e.g., LDLs, IDLs, VLDLs, Lp(a), chylomicrons) can be co-isolated, which is especially problematic for blood with high lipoprotein concentrations (Sódar et al., 2016; Karimi et al., 2018; Takov et al., 2019; Holcar et al., 2020). In our hands, in EV-enriched samples compared to plasma, SEC leads to a 100-fold reduction in ApoA1 concentrations (HDL marker) and a 50-fold reduction in ApoB100 concentrations (LDL, IDL, VLDL, Lp(a) marker) (Holcar et al., 2020). Such EV preparations are especially problematic when used for size and concentration determinations, as most techniques do not differentiate between the different nanoparticle types, such as EVs and lipoproteins. Care should also be taken when analyzing EV-bound miRNAs, as lipoproteins are known to transport RNA molecules, and also when performing functional assays, as lipoproteins can have biological effects (Vickers et al., 2011; Allen et al., 2018; Chiva-Blanch and Badimon, 2019; Freitas et al., 2019).

#### Ultrafiltration

In ultrafiltration, the use of membrane filters provides enrichment of EVs depending on their size in relation to the membrane pore size. In addition to centrifugal ultrafiltration, tangential flow filtration can also be used, where a sample does not flow through the membrane, but moves in a stream across the ultrafiltration membrane, in ‘tangential flow’ (Liangsupree et al., 2021). The isolation process of ultrafiltration can be influenced by the selection of filters with different pore sizes (e.g., 0.8, 0.45, 0.22, 0.1 µm) or of different materials (e.g., regenerated cellulose, stabilized cellulose, polyethersulfone, cellulose triacetate, anodic aluminum oxide, track-etched polycarbonate) (Vergauwen et al., 2017; Liangsupree et al., 2021). As several blood nanoparticles are similar in size to EVs, protein aggregates and lipoproteins are typically abundantly present in EV samples isolated from blood plasma by ultrafiltration (Ramirez et al., 2018). When isolating EVs from such complex biofluids, ultrafiltration is mainly used to concentrate down large volumes of sample in conjunction with other EV isolation methods, such as SEC or AF4 (Muller et al., 2014; Vergauwen et al., 2017; Dong et al., 2020). The chosen combination of methods affects the yields and characteristics of the EV isolates.

Unspecific binding of EVs to membranes used for ultrafiltration can also dramatically affect the yield of EV isolates. In a comparison of five different commonly used centrifugal filter types for their efficiency in concentrating recombinant GFP-labeled EVs spiked into phosphate-buffered saline, Vergauwen et al. (2017) investigated the membrane material (regenerated cellulose, Hydrosart, polyethersulfone, cellulose triacetate) and the pore size (10, 100 kDa). They concluded that regenerated cellulose membranes with a pore size of 10 kDa provided more than 100% recovery efficiency. Further, less than 40% recovery was achieved with the other filters, possibly due to binding of EVs to the membranes (Vergauwen et al., 2017).

As pressure or centrifugal force is commonly used to speed up ultrafiltration methods, EVs can also be deformed, or their size profiles can be shifted to smaller sizes. Finally, removal of all cells and cell remnants from blood plasma prior to ultrafiltration is necessary, as these can be similarly deformed into smaller particles when pushed through filter membranes, which can then be indistinguishable from physiological EVs.

#### Asymmetric Flow Field-Flow Fractionation

Particles can also be separated based on their diffusion coefficient using AF4, which has recently gained popularity for EV isolation (Sitar et al., 2015, 2017; Contado, 2017; Leeman et al., 2018; Oeyen et al., 2018; Zhang et al., 2018; Zhang and Lyden, 2019; Holcar et al., 2020; Wu et al., 2020). High-resolution separation (from a few nanometers up to micrometers) is achieved in a channel within a parabolic flow profile, against which a perpendicular cross-flow is applied. The particles are driven by the cross-flow toward the accumulation wall at the bottom of the channel, while also diffusing back into the channel because of the counteracting Brownian motion. Small particles with high diffusion coefficients float closer to the channel center and are displaced by the faster flow of the parabolic stream, whereas larger particles with smaller diffusion coefficients remain closer to the accumulation wall and are displaced by the slower flow. Thus, the particles are fractioned from smaller to larger sizes (Sitar et al., 2015; Contado, 2017). Importantly, due to the absence of a stationary phase, the separation in the channel is gentle and the surface area available for unwanted interactions is limited, which helps to preserve particle structure and avoid particle aggregation and loss (Wagner et al., 2014). However, blood nanoparticles of similar sizes to EVs can co-isolate, as seen for other methods of separation based on size. If AF4 is connected to a UV detector, protein aggregates and lipoprotein contaminants can be detected at 280 nm (Scheffer et al., 1997).

### Extracellular Vesicle Isolation Based on Particle Sedimentation Rate

#### Ultracentrifugation

Ultracentrifugation is the most commonly used method for isolating EVs (Furi et al., 2017). It involves centrifugation at high centrifugal forces (≥1,000,00× *g*) to separate particles depending on their sizes, shapes, and flotation densities. EV yields are dependent on the centrifugal force, rotor type (i.e., fixed angle vs. swinging bucket), pelleting efficiency (i.e., rotor and tube k-factors), and sample viscosity (Taylor and Shah, 2015). For example, different rotor pelleting efficiencies can result in different EV yields when isolating EVs from the same fluid, even when centrifuged for the same length of time (Witwer et al., 2013). Before ultracentrifugation, it is necessary to remove the cells, cellular debris, larger protein aggregates, and lipoproteins that differ in density to EVs, using differential centrifugation. This complex process leads to lower EV recovery, which can be additionally affected by the trapping of EVs by protein aggregates (Baranyai et al., 2015; Helwa et al., 2017). Repeated washing steps can reduce protein contaminants to some extent, but Western blotting indicates that albumin can remain in such EV preparations (Baranyai et al., 2015).

Another common contaminant in EV preparations after ultracentrifugation are HDLs, which are smaller but have similar flotation densities to EVs (Yuana et al., 2015). The purities can be improved by including additional separation through sucrose, iodixanol, or KBr density gradients, although these can further reduce the yield of EV preparations (Onódi et al., 2018). Specifically, ultracentrifugation separates low-density EVs (1.10–1.19 g/ml) from other particles with similar sedimentation coefficients, such as protein and RNA aggregates; however, HDLs (with densities 1.063–1.21 g/ml) can still be co-isolated (Lenassi et al., 2010; Yuana et al., 2014; Taylor and Shah, 2015; Konoshenko et al., 2018; Ramirez et al., 2018).

### Extracellular Vesicle Isolation Based on Solubility

Polymer polyethylene glycol (PEG), protein organic solvent precipitation (PROSPR) plus cold acetone or acetate-based isolation can be used to precipitate EVs based on removal of water; alternatively, protamine is a positively charged molecule used to form aggregates with negatively charged EVs (Deregibus et al., 2016; Gámez-Valero et al., 2016). Such precipitation methods are very unspecific for EV isolation as they can co-precipitate protein and RNA aggregates, lipoproteins, and any other nanoparticles with similar properties to EVs (Helwa et al., 2017; Konoshenko et al., 2018). The precipitating agent also becomes another potential contaminant that remains in the EV preparations, and therefore this method has a limited choice for downstream analysis. Precipitation is mainly used coupled to other EV isolation methods in connection with RNA characterization, or on low sample volumes (Gardiner et al., 2016). However, care needs to be taken when interpreting the results (Ramirez et al., 2018; Zhang et al., 2020). Several commercial kits are available, with differences reported for the yields, size distributions, and purities of the precipitated EVs (Patel et al., 2019).

### Extracellular Vesicle Isolation Based on Specific Markers

The presence of specific transmembrane proteins, receptors, and lipids on the surface of EVs allows for the isolation of EV subpopulations using immunoaffinity, which is based on strong and specific interactions between EV-specific antigens and related antibodies (Witwer et al., 2013). Most commonly, antibodies are bound to beads or other matrices, and they recognize different tetraspanins (e.g., CD9, CD63, CD81) or phosphatidylserine on the surface of EVs (Geeurickx et al., 2019; Martin-Jaular et al., 2021). Alternatively, negative selection for immune-depletion of unwanted components can be used before other EV isolation steps, which can remove the highly abundant blood proteins and lipoproteins (Mørk et al., 2017). Immunoaffinity separation is recommended for highly specific separation of EV subpopulations that carry characteristic markers (Witwer et al., 2013; Ramirez et al., 2018; Chen J. et al., 2019). Contaminants can be efficiently removed from such EV isolates, although broad knowledge of the EVs under study is needed, as otherwise information can be lost by the removal of EVs that do not carry the specific marker. The binding of antibodies to target EVs is also very strong, so their removal from the captured EVs is difficult, with the risk of adversely affecting the EVs in the process.

### Emerging Methods for Extracellular Vesicle Isolation

The more recently emerged microfluidics-based techniques show promise for an essential role in isolation, detection, and analysis of plasma EVs in the future (Guo et al., 2018). These are based on trapping EVs in microchannels, where the isolation, detection, and analysis of the EVs occur on a single integrated circuit of only a few square centimeters of a chip (Han et al., 2020). Microfluidics allow the processing of samples with very low volumes, thus also reducing the consumption of reagents. More importantly, even though microfluidics methods integrate multiple functional modules, they can be automated, which can provide high throughput and precision with short processing times (Chen YS. et al., 2019). Despite these advantages, the isolation step in microfluidics is often based on the same EV characteristics as described for classical isolation methods (i.e., surface biomarkers, size), and therefore these methods share the same limitations in terms of contaminants.

To overcome this, new methods have been developed. The use of devices with external force, such as electrophoretic or acoustic forces, can provide label-free isolation of EVs with relatively high purities and yields (Wu et al., 2017; Shi et al., 2019). Same applies to devices without external force, such as dynamic lateral displacement with nanopillars and viscoelastic inertial flow. However, both approaches demand high costs and precise operating conditions (Salafi et al., 2019). Recently, aqueous two-phase systems with bulky centrifugation have been used to separate EVs with high yields and moderate purities (Han et al., 2020). For aqueous two-phase systems on-chip, these are formed by dissolving two incompatible polymers, or a polymer and a salt, in water in a microfluidic channel, which can provide quick separation of EVs from proteins, and can be followed by precise collection at the outlet by laminar flow separation (Han et al., 2020).

These on-chip EV isolation methods can be followed by conventional detection and analysis of the EV isolates, or can be coupled to downstream microfluidics-based EV characterization (Guo et al., 2018; Lu et al., 2019), while retaining the same limitations in terms of the remaining contaminants. Integration of a microfluidics chip that lyses EVs using a surface acoustic wave, with a concentration and sensing microfluidics chip and an electrokinetic membrane sensor, recently showed potential for absolute quantification of both free-floating miRNAs and EV–miRNAs in plasma (Ramshani et al., 2019). Moreover, a microfluidics system that combines a membrane-based filtration module with a magnetic-bead-based immunoassay provided automated EV isolation and characterization directly from whole human blood (Chen YS. et al., 2019).

Integration of EV isolation with on-chip analysis also has great potential in cancer diagnosis and for monitoring of treatment responses, although standardized procedures for sample collection, storage, and pre-treatment, as well as the positive and negative criteria for the tested biomarkers, need to be determined before this can be fully translated into theranostics (Lu et al., 2019; Soekmadji et al., 2020). Moreover, the production of such chips is still very complex and expensive. At the moment, the lack of inexpensive, simple to use, scalable, and robust methods for the production of microfluidics devices is preventing the rapid mainstream adaptation of these technologies. However, in the future, microfluidics systems combined with viscoelastic fluids, optics, and plasmonics should provide opportunities for automated, transportable, precise, and high-throughput EV research, particularly when combined with the potential to be integrated with a smartphone or with machine-learning tools (Meng et al., 2021).

In summary, EVs can be isolated from blood using diverse techniques, each of which has its own advantages and disadvantages. A good understanding of possible contaminants in relation to EV isolation methods is thus needed for the appropriate selection of the downstream EV analysis methods and data interpretation. Combining two or more methods that can separate particles based on different EV characteristics is ideal to obtain pure EV preparations (Geeurickx et al., 2019; Zhang et al., 2020; Elgamal et al., 2021; Ji et al., 2021), although this can lead to increased losses of EVs, and is constrained by typically low starting volumes of biological samples. Advances in microfluidics hold great potential for rapid isolation of EVs from very small sample volumes; however, further improvements and validation are needed for clinical applications (Konoshenko et al., 2018; Chen J. et al., 2019; Ji et al., 2021; Pasetto et al., 2021).

## Interpretation of Downstream Characterization Methods in Blood Biomarker Studies Depends on the Purity of the Extracellular Vesicle Preparations

Extracellular vesicle characterization methods can generally be divided into biochemical methods, which help to identify the nucleic acid, protein, lipid, and metabolite compositions of EVs, and biophysical methods, which help to describe EVs according to size, concentration, charge, density, stiffness, and light scattering. Better characterization of EVs is important to understand their fundamental roles in physiological and pathological processes, and this knowledge also needs to be translated into the clinic to be used for improved diagnostics and therapies. To improve the interpretation of EV functional studies and the reliability and reproducibility of EV biomarker studies, an understanding of the influence of blood contaminants on EV characterization is needed. The first step is to understand the main principle of analysis for each technique and if it can account for the contaminants that are expected to be present in the samples after the preceding EV isolation method.

### Biochemical Methods for Identification of the Molecular Composition of Extracellular Vesicles

Most EV studies are interested in their nucleic acid and protein compositions, although there is increasing interest also for their lipids and metabolites (Gassart et al., 2003; Lydic et al., 2016; Ditiatkovski et al., 2020). The molecular composition of EVs is dependent on the type of the cell or tissue of origin, and also reflects their (patho)physiological state. This can help to identify origin-specific subsets of EVs in the blood, and to detect EV molecular signatures related to diseases. Therefore, blood EVs are a very promising source of biomarkers for diverse diseases.

Extracellular vesicle-associated RNAs (most commonly miRNAs, but also long noncoding RNAs, viral RNAs) represent one of the most promising and frequently studied EV-related biomarkers, as these have important roles in disease etiology and pathogenesis, and are present in sufficient quantities for detection by established molecular methods (Amorim et al., 2017; Mateescu et al., 2017; Tang et al., 2017; Soekmadji et al., 2018; Profiling et al., 2019; Srinivasan et al., 2019; Driedonks et al., 2020; Hooten, 2020; Liang et al., 2020). Much less is known about EV-associated DNA, although it appears that the DNA can be located in the EV lumen or attached to the surface of EVs as single or double-stranded molecules that are protected from degradation by the bound histones. EV-associated DNA is also heterogeneous in origin (i.e., genomic, mitochondrial) and size (i.e., a few hundred base pairs in small EVs, up to >2 million base pairs in large EVs) (Goričar et al., 2021). EVs protect the nucleic acids from degradation by nucleases that are commonly present in biological fluids, making them remarkably stable under different storage conditions.

To gain unbiased knowledge, next-generation sequencing or PCR profiling arrays have been used to analyze nucleic acids extracted from EVs, while quantitative PCR is used to test interesting targets on larger cohorts (Soekmadji et al., 2018). As changes in EV-enclosed RNA compositions due to diseases can be relatively small, it is important to remove any extra-vesicular RNAs that might be bound to co-isolated contaminants (Gallo et al., 2012; Bracht et al., 2021). Protein and lipoprotein contaminants are known to carry miRNAs in the blood, and might therefore alter EV RNA analysis (Vickers et al., 2011; Vergauwen et al., 2017; Buzás et al., 2018). Before RNA analysis, EV preparations can thus be processed using proteinases and RNases to remove extra-vesicular RNA (Bracht et al., 2021). If platelet-derived EVs are also present due to improper collecting and processing of the blood, then these can overshadow the pathology-dependent miRNA signals (Palviainen et al., 2020). Cell-free DNA analysis, on the other hand, is mostly constrained by the low quantity and fragmentation of EV-bound DNA, and is not particularly affected by blood contaminants (Zhang L. et al., 2019; Keller et al., 2021).

Extracellular vesicle-associated proteins are another promising source of biomarkers, as these can have many important roles in disease pathologies. Surface proteins can be used for enrichment of EVs that carry specific markers, or for direct quantification of specific EV subpopulations, which overcomes the problem of dilution of pathology-related EV signals in the blood. For quantification of specific protein targets, Western blotting or ELISA are most often used, while for unbiased characterization of EV-associated proteins, mass spectrometry and EV flow cytometry (described in Section 4.2) are generally used (Pocsfalvi et al., 2016; Görgens et al., 2019; Tian et al., 2020; Martin-Jaular et al., 2021). The presence of EVs in a sample can often be confirmed using Western blotting of common EV-related proteins, such as tetraspanins, Alix, Tsg101, and HSP70. As most EV markers are also detectable in whole cells, sample purity should be assessed by EV-negative controls, such as calnexin or histones (Ramirez et al., 2018). ELISA can provide relatively cheap detection of target proteins in a large number of samples, and it is therefore well established and widely used in research and medical applications (Pocsfalvi et al., 2016).

Commercial EV protein detection platforms and kits are now becoming available to simplify EV protein analysis. Commercial ELISA kits can help with quantification of generic EV-positive markers, such as tetraspanins, or disease-related membrane proteins, like PD-L1 or ICAM-1, or EV luminal cargo, like cytokines (Nardi et al., 2016; Hosseinkhani et al., 2017; Hosseinkhani et al., 2020; Venkatesan et al., 2017; Willis et al., 2017; Cordonnier et al., 2020). ELISA can also be used to measure the level of contaminants in a sample (e.g., apolipoproteins, albumin) (Nardi et al., 2016; Holcar et al., 2020). Other antibody-based methods include phenotyping of EVs using diverse antibody-coated bead technologies (e.g., flow cytometry bead-based multiplex analysis, such as ‘MACSPlex’ technology; multiplex bead-based immunoassays, such as ‘Luminex’ technology) or antibody-coated surfaces (e.g., EV protein arrays; multiplexed microarray chips for the immuno-capture of EVs, such as ‘ExoView’ technology) (Rausch et al., 2016; Bachurski et al., 2019; Brahmer et al., 2019; Štok et al., 2020). Users can usually decide between predetermined combinations of antigens included in a test (as usually CD9, CD63, CD81), or they can obtain a custom combination of chosen targets.

Rapid growth of mass-spectrometry-based strategies for proteome characterization has in recent years improved the level of molecular details that can be obtained from limited amounts of EVs isolated from blood (Rosa-Fernandes et al., 2017). The basic workflow for EV proteome determination using mass spectrometry is based on tryptic digestion of the extracted protein mixture, followed by separation of proteins by nano high-pressure liquid chromatography and detection by tandem mass spectrometry (i.e., ‘nanoHPLC-MS/MS’). This technique can identify thousands of proteins in a mixture, although distinguishing rarer proteins of interest from the background of highly abundant proteins can be a real challenge (Ruhen and Meehan, 2019). In all of these mentioned methods, protein and lipoprotein contaminants of EV isolates would directly affect the EV protein analysis, and therefore these need to be removed or allowed for. The latter can be achieved by co-analysis of lipoprotein- and protein-depleted samples during the method development, thus defining the contribution of the contaminants to the final results, or by quantifying the (lipo)protein concentrations in EV samples using simple methods such as ELISA.

According to MISEV 2018, albumin and apolipoproteins are the best markers of EV isolate contamination (Théry et al., 2019). To account for contamination with blood (lipo)proteins, albumin and ApoA1 (for HDLs) and ApoB (for LDLs, IDLs, VLDLs, Lp(a) and chylomicrons) levels can be determined by ELISA or Western blotting (Baranyai et al., 2015; Hong et al., 2016; Pietrowska et al., 2019; Gaspar et al., 2020; Holcar et al., 2020). To remove proteins, EV preparations can be processed using the proteases proteinase K, trypsin, and others, although this can impact upon the availability of any surface antigens, and thus impact upon the EV analysis (Muller et al., 2014; Shelke et al., 2014; Cvjetkovic et al., 2016; Stewart et al., 2016; Chettimada et al., 2018; Skliar et al., 2018; Choi et al., 2020).

### Biophysical Methods for Characterization of Extracellular Vesicle Size and Concentration

Certain pathological factors can induce changes in EV size profiles and concentrations, such as hypoxia, autophagy, and stress (all typical of cancers). Thus, these biophysical characteristics of EVs can also be evaluated as biomarkers of disease states. Additionally, the sizes, charge, and membrane stiffness of EVs can affect their interactions with other particles or membranes, and are therefore important for the functional roles of EVs (Zhang et al., 2018).

#### Nanoparticle Tracking Analysis and Dynamic Light Scattering

These techniques both combine laser-light scattering of the particles in the sample for the measurement of the concentration and size distributions (Filipe et al., 2010). They are also both dependent on the Brownian movement of nanoparticles in solution. Nanoparticle tracking analysis (NTA) measures this movement through tracking and analysis of the particles under the microscope, and detection using a charge-coupled device camera for the amount of laser light refraction on a particle-by-particle basis. The movement of the nanoparticles is related to their size, with the refractive index of the nanoparticles as the limiting factor for detection. NTA can be used to detect particles with a hydrodynamic diameter of 30–1,000 nm (Chernyshev et al., 2015).

Dynamic light scattering (DLS) records time-dependent fluctuations in the scattered light intensity caused by interference from the nanoparticles in a sample, without visualizing the nanoparticles. It measures all of the particles in a suspension in the size range of 5–6,000 nm at the same time. The reported average particle size is thus biased toward the larger particles within a sample, as these scatter light more intensely than the smaller particles. DLS can also measure the charge that develops at the interface between the surface of EVs and their liquid medium, which is known as the zeta potential. The zeta potential of EVs is usually from -9 mV to -16 mV (Zhang et al., 2018). DLS is very effective for the analysis of homogeneous solutions of very small particles, whereas particle-by-particle measurement using the NTA approach is better for polydispersed samples, although larger particles can hinder detection of EVs in the sample by saturation of the camera, which will distort the results (Palmieri et al., 2014; Erdbrügger et al., 2016). As NTA and DLS detect particles only indirectly through refracted laser light, contaminants of similar sizes to the EVs will be indiscriminately detected and measured along with the EVs, such as protein aggregates, immune complexes or lipoproteins, thus effecting the analysis (Mørk et al., 2017). NTA and DLS should thus ideally follow EV isolation techniques that minimize contamination of EVs with particles in the same size range as EVs, or larger. Lipoproteins other than HDLs, which are too small to be detected by these methods, can be separated from EVs by ultracentrifugation combined with a density gradient/cushion (e.g., sucrose, iodixanol, KBr), based on the differences in their densities (Yuana et al., 2014; Sódar et al., 2016; Karimi et al., 2018). Larger protein aggregates can be removed with mild proteolytic treatments, although the potential effects of such treatments on the perceived EV size and bioactive EV-protein corona should be considered (Cvjetkovic et al., 2016; Skliar et al., 2018; Choi et al., 2020).

#### Resistive Pulse Sensing

Resistive pulse sensing (RPS) is a method that can be used to determine EV size distributions and concentrations, with detection here based on a transient change in an electric signal (Witwer et al., 2013). The system consists of two fluid cells that are filled with a conductive liquid, and that contain the sample. The fluid cells are separated by a nonconductive membrane with a single pore, which is under a baseline electric current. As a particle passes through the stretchable nanopore under the electric current, the current is transiently attenuated in proportion with the particle volume. As the detection range of specific particle sizes depends on the specific type of nanopore and the stretch that is applied to it, the use of various nanopore membranes is required to detect the entire EV size range when considering measurements of polydispersed EV populations with heterogeneous particle sizes (Witwer et al., 2013; Mørk et al., 2016; Ramirez et al., 2018). In some samples, pore-clogging can occur, which is another disadvantage of RPS (van der Pol et al., 2014). Like NTA, at the current stage, RPS does not provide particle phenotyping and discrimination between EVs and contaminants, such as protein aggregates or lipoproteins (Mørk et al., 2016).

#### Flow Cytometry

Flow cytometry is a technique that combines and uses principles of biochemical methods for the identification of the molecular compositions of EVs, and biophysical methods for characterization of EV sizes and concentrations. In classical flow cytometry, EVs can be labeled with fluorescent dyes and/or antibodies (e.g., against tetraspanins or a specific protein of interest). The samples are then passed through multiple lasers of differing wavelengths, with the scattered light detected. The amounts and directions of the forward scatter (i.e., ‘FSC’) or the fluorescence indicates the size of the EVs measured, while the side scatter (i.e., ‘SSC’) indicates the internal complexity of the observed particle (Gandham et al., 2020). Flow cytometry itself is a high-throughput, single-particle, multi-parameter analysis technique that can be used to analyze a large range of particle diameters (McVey et al., 2018).

The major problems and restrictions of flow cytometry for characterization of EVs do not arise as a result of contaminations of the EV samples; they are predominantly a consequence of technical limitations of the method itself. These include standardization of the light scattering and fluorescence data between different flow cytometers, limited resolution and detection of EVs <200 nm in diameter, the possibility of unspecific staining, and low brightness of the fluorochromes coupled to antibodies (Morales-Kastresana et al., 2017; McVey et al., 2018). This has led to the development of alternative approaches to improve small EV detection by flow cytometry, including nanoflow cytometry, high-resolution cytometry, and fluorescent triggering and imaging flow cytometry (Freitas et al., 2019; Görgens et al., 2019; Tian et al., 2020).

Similar to other techniques, another challenge of flow cytometry is the adequate and transparent reporting of the findings and the ambiguities inherent in the data interpretation (Welsh et al., 2017; Welsh et al., 2020a; Welsh et al., 2020b; Ramirez et al., 2018; Görgens et al., 2019; Tang et al., 2019; Ender et al., 2020). This was addressed in 2020 by a position paper on the minimum information that should be provided in an EV flow-cytometry-specific reporting framework (i.e., ‘MIFlowCyt-EV’). This publication defined the critical information that should be reported relating to experimental design, sample staining, and EV detection and measurement in studies that report EV flow-cytometry data. Standardized reporting can improve quantitative comparisons of results from different laboratories, and support the development of new instruments and assays (Welsh et al., 2020a). For deeper insight into EV flow cytometry, please also consult the following articles (Arraud et al., 2016; Welsh et al., 2017; Welsh et al., 2020a; van der Pol et al., 2018; Görgens et al., 2019; Tian et al., 2020; Yang and Rhee, 2021).

#### Transmission Electron Microscopy and Scanning Electron Microscopy

These electron microscopy techniques can be used for morphological and structural characterization of EVs, and for EV purity evaluation and contaminant identification (Cvjetkovic et al., 2016; Gámez-Valero et al., 2016). The methods are based on the detection of interactions between the electrons in fixed samples and those in the beam of accelerated electrons, which are transmitted through the sample in transmission electron microscopy (TEM), and over the surface of the sample in scanning electron microscopy (SEM). Differentiation of the edges and features of the sample depends primarily on the differences in the electron densities of different organic molecules in the samples. In electron microscopy, EVs can be observed as unstained, or can be stained to provide higher electron densities. Staining can be unspecific (e.g., osmium tetroxide, uranyl acetate, phosphotungstic acid) or specific (e.g., immunoTEM), with gold-labeled antibodies (Brisson et al., 2017). Protein aggregates have different electron densities than EVs after staining, which makes their presence in the sample very apparent (Muller et al., 2014; Hong et al., 2016; Karimi et al., 2018). Similarly, the presence of a clear phospholipid bilayer distinctly separates EVs from lipoproteins, even when they are of comparable sizes and shapes (Chernyshev et al., 2015; Sódar et al., 2016). However, the fixation and desiccation steps required for TEM and SEM can lead to nonuniform drying fronts, which can alter the sizes of EVs, and result in shape distortion (e.g., their characteristic ‘cup-shaped’ morphology) (Chernyshev et al., 2015). A special modification of classic TEM is provided by cryogenic (cryo-)TEM, which allows for direct observation of EVs without the dehydration, chemical fixation, and/or staining (Murata and Wolf, 2018). While cryo-TEM is particularly labor-intensive and requires a skilled operator, freezing of samples in their native hydrated state ensures the conservation of the physiological volume of the EVs. Capturing multiple dynamic states of EVs also allows three-dimensional tomography and spatial visualization of more complex structures in samples (Chernyshev et al., 2015; Murata and Wolf, 2018; Sharma et al., 2018). The maximum size of an observed object in cryo-TEM is limited by the thickness of the sample that can still be penetrated by the electron beam, which is ∼500 nm in the case of 300 kV TEM (Murata and Wolf, 2018).

#### Atomic Force Microscopy

Atomic force microscopy (AFM) is an emerging alternative to optical and electron diffraction methods for studying EVs. In AFM, a very fine tip of a probe (i.e., a few nanometers) is scanned over a sample, in raster, line-by-line. The height of the tip is adjusted based on the instrument feedback, which translates into mechanical information and the topography of the sample. This feedback to the instrument can come from the frequency or amplitude of the tip oscillation, and the force exerted on the cantilever by the surface, or some combination of these three (Sharma et al., 2018). AFM primarily explores the mechanical properties of a sample. In the case of EVs, it analyzes the stiffness of the surface of the EVs, which has been shown to be dependent on their (patho)physiological state (Parisse et al., 2017). AFM can simultaneously measure the distribution of EV sizes and map their stiffness with nanometric precision. An antibody-coated tip and surfaces (e.g., mica, glass) can also be used to examine subsets of EVs, or to functionally analyze the morphology and structural heterogeneity of EVs (Parisse et al., 2017; Sharma et al., 2018).

Similar to electron microscopy, AFM is not a method that is suited for the analysis of large numbers of samples, or for conducting statistical analysis of an EV population; however, it provides accurate analysis of individual vesicles and their surfaces. Isolation methods that can significantly alter the surface or even sizes of EVs can affect these analyses. Evident protein contamination, aggregation of the sample, use of immunoprecipitation as the method of isolation, or any treatments with enzymes can all alter the outer surface of EVs (including the protein corona), and therefore these should be carefully considered when interpreting the results (Woo et al., 2016)*.*

To summarize, biochemical and biophysical EV analysis methods are in general affected by any remaining nanosized contaminants in the EV isolates, although these effects can be minimized by pairing the EV characterization analysis with compatible EV isolation method(s) in terms of potential contaminants. Alternatively, EV analysis data should be interpreted carefully, taking into account any influence on the experiment outcomes of contaminants that might be present. Deeper understanding of the EV analysis methods and how they are influenced by specific contaminants will help to improve the interpretation of EV biomarker studies, and thereby their reliability and reproducibility.

## Conclusion and Future Perspectives

Extracellular vesicles are membrane-bound nanometer-to micrometer-sized particles, which in humans are released from all cells and can accumulate in the blood and other body fluids. The sizes, concentrations and molecular compositions of EVs reflect the type and state of their cell of origin, making them intriguing candidates for research and biomarker discovery (Holcar et al., 2020; Palviainen et al., 2020). Peripheral blood is easily accessible, and is thus one of the most desirable sources of biological EVs. However, blood is a very complex fluid that has an abundance of nanoparticles that share molecular or/and biophysical characteristics with EVs, and can therefore co-isolate from blood plasma. This can obscure the biomarker potential or biological relevance of EVs, thus also distorting the outcomes of biomarker studies. However, most biomarker studies do not address the problem of contaminants in EV isolates, and how they can affect the downstream analysis.

To the best of our knowledge, this review is the first comprehensive description of the effects of different EV isolation methods on the co-isolation of major contaminants from blood plasma, which also covers the effects that these contaminants can have on downstream EV analysis. To account for co-isolated contaminants and improve the interpretation of these biomarker studies, a good understanding of the main principles of analysis for each technique is also needed. We believe that the details provided and discussed here will help to improve the rigor, reproducibility, and reliability of future EV biomarker studies.

Despite high expectations, investment of considerable effort, and promising results, EV-related biomarkers have not yet been routinely implemented in clinical practice, and much of the basic EV research remains difficult to reproduce (Popovic et al., 2018; Elgamal et al., 2021). An important step to overcome this is to consciously strive for rigor in the standardization of methods used in the field, with special care dedicated to the preanalytical stages in particular (Théry et al., 2019; Welsh et al., 2020a; Nieuwland et al., 2020). Consistent quantification of nonEV contaminants, thorough and transparent reporting of protocols used (preferably using ISEV-recommended platforms, such as EV-TRACK), and use of EV-reference materials will also facilitate better inter-study comparisons and help to provide more reliable and nuanced interpretations of results obtained (Van Deun et al., 2017; Geeurickx et al., 2019; Görgens et al., 2019; Tang et al., 2019). Furthermore, the inherent heterogeneity of EVs should be further addressed by improvements to the existing methods for EV isolation and characterization, and by development of new methods or creative combinations of those already established (Chang et al., 2021; Ji et al., 2021; Martin-Jaular et al., 2021). Methods that can provide uniform subpopulations of EVs of high purities to which (sub)cellular origins can be attributed and for which functions can be defined will help to further the field and realize the full potential of EVs in the research and clinical settings.
